# Increased Diagnostic Yield by Reanalysis of Whole Exome Sequencing Data in Mitochondrial Disease

**DOI:** 10.3233/JND-240020

**Published:** 2024-07-02

**Authors:** Catarina Olimpio, Ida Paramonov, Leslie Matalonga, Steven Laurie, Katherine Schon, Kiran Polavarapu, Janbernd Kirschner, Ulrike Schara-Schmidt, Hanns Lochmüller, Patrick F. Chinnery, Rita Horvath

**Affiliations:** a Department of Clinical Neurosciences, John Van Geest Centre for Brain Repair, University of Cambridge, Cambridge, UK; b East Anglian Medical Genetics Service, Cambridge University Hospitals NHS Foundation Trust, Cambridge, UK; c Centro Nacional de Análisis Genómico, Barcelona, Spain; d Department of Neuropediatrics and Muscle Disorders, Faculty of Medicine, Medical Center-University of Freiburg, Freiburg, Germany; e Department of Pediatric Neurology, Center for Neuromuscular Disorders, Center for Translational Neuro- and Behavioral Sciences (C-TNBS), University Hospital Essen, Essen, Germany; f Children’s Hospital of Eastern Ontario Research Institute, Ottawa, ON, Canada; g Brain and Mind Research Institute, University of Ottawa, Ottawa, ON, Canada; h Division of Neurology, Department of Medicine, The Ottawa Hospital, Ottawa, ON, Canada; i MRC Mitochondrial Biology Unit, Department of Clinical Neurosciences, University of Cambridge, Cambridge, UK

**Keywords:** Mitochondrial diseases, respiratory chain deficiencies, next generation sequencing, mitochondrial genes

## Abstract

**Background:**

The genetic diagnosis of mitochondrial disorders is complicated by its genetic and phenotypic complexity. Next generation sequencing techniques have much improved the diagnostic yield for these conditions. A cohort of individuals with multiple respiratory chain deficiencies, reported in the literature 10 years ago, had a diagnostic rate of 60% by whole exome sequencing (WES) but 40% remained undiagnosed.

**Objective:**

We aimed to identify a genetic diagnosis by reanalysis of the WES data for the undiagnosed arm of this 10-year-old cohort of patients with suspected mitochondrial disorders.

**Methods:**

The WES data was transferred and processed by the RD-Connect Genome-Phenome Analysis Platform (GPAP) using their standardized pipeline. Variant prioritisation was carried out on the RD-Connect GPAP.

**Results:**

Singleton WES data from 14 individuals was reanalysed. We identified a possible or likely genetic diagnosis in 8 patients (8/14, 57%). The variants identified were in a combination of mitochondrial DNA (*n* = 1, *MT-TN*), nuclear encoded mitochondrial genes (*n* = 2, *PDHA1*, and *SUCLA2*) and nuclear genes associated with nonmitochondrial disorders (*n* = 5, *PNPLA2, CDC40, NBAS* and *SLC7A7*). Variants in both the *NBAS* and *CDC40* genes were established as disease causing after the original cohort was published. We increased the diagnostic yield for the original cohort by 15% without generating any further genomic data.

**Conclusions:**

In the era of multiomics we highlight that reanalysis of existing WES data is a valid tool for generating additional diagnosis in patients with suspected mitochondrial disease, particularly when more time has passed to allow for new bioinformatic pipelines to emerge, for the development of new tools in variant interpretation aiding in reclassification of variants and the expansion of scientific knowledge on additional genes.

## INTRODUCTION

Mitochondrial disorders are highly heterogeneous and complex both in their phenotype and genotype [1]. They are individually rare but collectively affect 1 in 5000 individuals. Mitochondria are well known for their role in oxidative phosphorylation and ATP synthesis and both mitochondrial DNA (mtDNA) and nuclear DNA (nDNA) encoded genes contribute to this key mitochondrial function. Mitochondrial disorders can present with a broad range of symptoms affecting virtually every organ system. This complexity in genotype and phenotype is reflected in the difficulties and burden reported by patients, that often see multiple doctors over many years before a diagnosis is established [2]. It is also reflected in the difficulty in establishing a genetic diagnosis in patients with a suspected mitochondrial disorder.

Traditionally, mitochondrial disorders have been diagnosed primarily by a combination of clinical features and histochemical or biochemical analysis of an affected tissue, followed by targeted molecular genetic testing [3]. With Next Generation Sequencing (NGS), exome and genome sequencing became cost-effective, and the diagnosis of rare diseases, including mitochondrial disorders, was transformed. In recent years these technologies have been used exponentially in the diagnosis of mitochondrial diseases. In cohorts of patients with suspected mitochondrial disorders whole exome sequencing (WES) establishes a diagnosis in 40–60% of the patients [4–7]. Crucial to a high yield in genetic diagnoses is a well-established phenotype.

Several methods have been proposed to increase the diagnostic yield established by WES for mitochondrial disorders, including whole genome sequencing, suggested as the ideal first line genomic investigation [8], long read sequencing and Deep-WES as well as using other ‘omics’ such as RNA sequencing, epigenomics and metabolomics [9]. However, not identifying a causative variant from WES does not necessarily mean the disease-causing variant lies outside the data already produced and reanalysis of WES is a valuable approach to further increase diagnostic yield [10].

Here we proposed to reanalyse the WES data of a well phenotyped cohort of patients with a suspected mitochondrial disease, with combined respiratory chain deficiency, initially reported 10 years ago [4], using the improved WES diagnostic pipeline provided by the RD-Connect Genome-Phenome Analysis Platform (GPAP).

The RD-Connect GPAP (https://platform.rd-connect.eu) is a user-friendly resource of genomic and phenotypic data of patients with rare disease and family members, which facilitates diagnosis and gene discovery, and has been used as the primary analysis tool in a number of large European projects [11].

## MATERIALS AND METHODS

### Original cohort

Taylor and colleagues [4] compiled a cohort of 53 patients with suspected mitochondrial disease and histochemical and/or biochemical evidence of decreased activities of multiple respiratory chain complexes in a clinically affected tissue based on published criteria [12]. These patients were previously tested for and did not have large scale mtDNA rearrangements, mtDNA depletion, and mtDNA point mutations (with the exception of some patients, including patient 5 in our cohort, in whom decreased levels of mtDNA was confirmed in muscle).

In the original Taylor *et al* cohort the variants were called by an in-house pipeline and classified into 4 groups: 1) presumptive pathogenic –homozygous or compound heterozygous mutations in genes previously shown to cause multiple respiratory chain deficiencies; 2) possible pathogenic –homozygous or compound heterozygous mutations in novel genes predicted to case a mitochondrial translation defect based on their proposed function and similarity to known disease genes; 3) variants of unknown significance –homozygous or compound heterozygous mutations in novel or known disease genes not known to be associated with mitochondrial pathology and; 4) unresolved cases in which a single plausible genetic cause could not be identified [4]. They established a probable or possible diagnosis in 32 of 53 patients (60%). Despite the high diagnostic yield, an additional 21 patients did not have a diagnosis established following careful biochemical phenotyping and whole exome analysis.

### Our cohort

**Fig.1:**
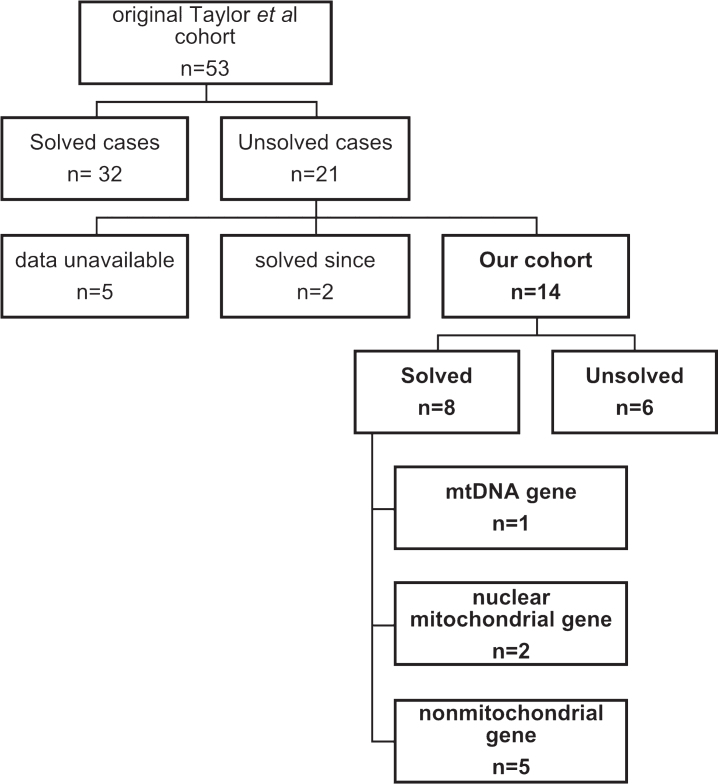
Patient cohort and number of diagnosed and undiagnosed patients

We collated the 21 patients with no established diagnosis in the original cohort including patients with variants of uncertain significance. For 5 of the 21 patients the consent forms did not allow the upload of data to the RD-Connect GPAP platform. Two of the 21 patients were since found to have disease causing variants, one in MIEF2 [13] and another one in TANGO2 [14]. Fourteen of the 21 patients had, to our knowledge, no genetic cause identified for their condition (Fig. 1).

### RD-Connect GPAP

The RD-Connect GPAP is a user-friendly online resource which facilitates the collation, analysis, interpretation and sharing of integrated genome-phenome datasets. Phenotypic and raw WES data from our cohort was submitted to RD-connect GPAP [11].

### Whole exome sequencing and data analysis

Exome sequencing was performed on whole blood tissue samples in Newcastle upon Tyne as previously described [4]. The raw WES data, excluding the 5 cases without the appropriate consent forms, was since transferred and processed by the RD-connect GPAP using their standardized pipeline (as described in Laurie et al. [11]).

Variant prioritisation was carried out on the RD-Connect GPAP. Likely pathogenic variants were identified by applying standard filtering criteria including moderate to high variant effect predictor (VEP) score (i.e. nonsense, splice site, frame-shift, in-frame and non-synonymous variants), gnomAD allele frequency of <0.01 and a GPAP internal frequency <0.02. WES data was sequentially analysed on GPAP using available gene lists on the platform (such as Mitocarta 2.0, *n* = 1158; European Reference Network on Neurological Diseases (ERN-RDN) *n* = 1820 and on Neuromuscular Diseases (ERN-NMD), Muscle Gene Table May 2021, *n* = 611, Medically Interpretable Genome, *n* = 5419, Genomics England Mitochondrial disorders panel v.4.157, *n* = 487; and Genomics England Inborn Errors of Metabolism panel v.4.129, *n* = 934). If no candidate variant was identified, we created patient specific phenotype-based gene lists using Human Phenotype Ontology (HPO) as embedded on GPAP and also searched for variants across the whole exome dataset.

Candidate variants were selected based on their predicted in silico deleteriousness, previous known association with human disease and phenotype fit (evaluated using OMIM and reviewing the literature in PubMed). Possible causative variants were individually classified according to the American College of Medical Genetics (ACMG) standards and guidelines for interpretation of variants [15] and ACGS Best Practice Guidelines for Variant Classification in Rare Disease [16]. For mtDNA variants the ACMG/AMP standards and guidelines for mitochondrial DNA variant interpretation [17] was used.

Due to the historical nature of the data set we were not able to do segregation studies or confirm phase for the presumed compound heterozygous variants.

## RESULTS

### Our cohort

The demographics and phenotype of the 14 patients included in our cohort is described in Table 1. Of note all patients in our cohort were children or young adults (<18 years of age) at the time of recruitment. Five were female (36%) and nine were male (64%).

**Table 1:** Patient cohort: phenotype and demographics

	Gender	Age at onset	Consanguinity	Affected organs	Histochemistry	Deficient complexes	Other
P1	F	2 yr	yes	Muscle, CNS	Normal	cI, cIV	–
P2	M	17 yr	no	CNS	COX deficiency	cI, cIV	Optic atrophy, axonal neuropathy
P3	F	7 yr	yes	Muscle, CNS	RRF, COX mosaic	–	Multiple mitochondrial deletions, PEO, raised CK
P4	M	4 yr	Yes	Muscle, CNS, Liver	COX mosaic	cI, cIV	Lactic acidosis, growth retardation
P5	M	2 wk	Yes	Muscle	COX mosaic	cI, cIV	Multiorgan failure and lactic acidosis
P6	M	Birth	No	Muscle, CNS, Heart	COX mosaic	cI, cIV	Raised CK
P7	M	6 yr	No	Muscle	COX mosaic, RRF and lipid	cI, cIII and cIV	Raised CK
P8	M	18 mo	unknown	Muscle, Liver	COX normal, SDH positive	cI, cIII	–
P9	F	1 mo	No	Muscle, CNS, Liver	COX mosaic	cI, cIV	mtDNA depletion, immune defect, deafness, renal failure
P10	M	6 yr	No	Muscle	RRF, COX mosaic	cI, cIV	PEO, lactic acidosis, raised CK
P11	M	2 mo	Yes	Muscle, CNS, Liver	COX mosaic	cI, cIV	Alpers’ phenotype
P12	F	1 yr	No	Muscle, CNS	COX normal, lipid storage	cI, cIII	Lactic acidosis
P13	M	2 yr	No	Muscle, CNS, Liver	COX normal, RRF	cIII, cIV	Lactic acidosis
P14	F	Birth	No	Muscle, CNS	Normal	cI, cIV	Lactic acidosis

F: female, M: male, Yr: years; wk: weeks; mo: months; CNS: central nervous system; RRF: raggered red fibres; cI: complex I; cII: complex II; cIII: complex III; cVI: complex VI; PEO: progressive external ophthalmoplegia; CK: creatinine kinase.

### Genetic results

Singleton whole exome sequencing (WES) data from the 14 individuals with suspected mitochondrial disease and multiple respiratory chain defects was reanalysed using the improved WES diagnostic pipeline provided by the RD-Connect GPAP.

Our reanalysis identified a possible or likely genetic diagnosis in a further 8 patients (8/14, 57%). The variants identified were a combination of mitochondrial DNA point mutations (*n* = 1, *MT-TN*), variants in nuclear encoded mitochondrial genes (*n* = 2, *PDHA1*, and *SUCLA2*) and variants in nuclear genes associated with other inborn errors of metabolism (*n* = 5, *PNPLA2, CDC40, NBAS* and *SLC7A7*). The details of each variant as well as their ACMG classification is described in Table 2. With the exception of *MT-TN* that follows mitochondrial inheritance and *PDHA1* which is X-linked, all show autosomal recessive inheritance (Table 3). In 6 patients, a possible or probable genetic diagnosis could not be established, including one patient who subsequently had trio whole genome sequencing and this data added to and analysed by our team on the RD-connect GPAP.

**Table 2:** Likely or possible genetic diagnoses identified in our cohort

	Gene	Genotype	Variant	ACMG classification	ACMG criteria	Variant previously reported
P3	*MT-TN*	Heteroplasmic (49%)	m.5698 G > A	VUS	PS3 supPM2 mod	Spinazzola et al., 2004
P4	*SLC7A7*	Homozygous	NM_003982.4:c.1A>Cp.Met1?	LP	PVS1 modPS1 modPS3 supPM2 mod	Sperandeo et al., 2008[30]
P7	*PNPLA2*	Presumedcompound heterozygous	NM_020376.4:c.24 G > Ap.Trp8Ter	P	PVS1 very strongPM2 mod	Pennisi et al., 2017[31]
			NM_020376.4:c.798dup p.Ala267ArgfsTer40	P	PVS1 very strongM2 modPM3 sup	Jousserand et al., 2016[32]
P8	*NBAS*	Presumedcompound heterozygous	NM_015909.4:c.2330C > Ap.Pro777His	VUS	PM2 modPP3 sup	Haack et al., 2015 [18]
			NM_015909.4:c.1187G > Ap.Trp396Ter	P	PVS1 strongPM2 mod	Haack et al., 2015 [18]
P11	*SUCLA2*	Homozygous	NM_003850.3:c.370T > C p.Ser124Pro	VUS	PM2 modPP3 mod	Issa et al., 2020[33]
P12	*NBAS*	Presumedcompound heterozygous	NM_015909.4:c.2330 C > Ap.Pro777His	VUS	PM2 modPP3 sup	Haack et al., 2015 [18]
			NM_015909.4:c.1187 G > Ap.Trp396Ter	P	PVS1 strongPM2 mod	Haack et al., 2015 [18]
P13	*PDHA1*	Hemizygous	NM_000284.4:c.905 G > Ap.Arg302His	P	PM1 modPM2 modPM5 modPP3 strong	Otero et al., 1998[34]
P14	*CDC40*	Homozygous	NM_015891.3:c.1505T > G p.Phe502Cys	LP	PS3 modPM2 modPM3 supPP2 strong	Chai et al., 2021[35]

P: pathogenic; LP: likely pathogenic; VUS: variant of uncertain significance.

**Table 3:** Causative genes in our cohort and associated phenotypes

Genes	OMIM #	Associated OMIM Phenotype
*Mitochondrial disease*
*MT-TN*	* 590010	Mitochondrial Complex I deficiency; Isolated opthalmoplegia
*SUCLA2*	* 603921	AR Mitochondrial DNA depletion syndrome 5
*PDHA1*	* 300502	XLD Pyruvate dehydrogenase E1-alpha deficiency
*Other inborn errors of metabolism*
*PNPLA2*	* 609059	AR Neutral lipid storage disease with myopathy
*NBAS*	* 608025	AR Infantile liver failure syndrome 2AR Short stature, optic nerve atrophy, and Pelger-Huet anomaly
*SLC7A7*	* 603593	AR Lysinuric protein intolerance
*CDC40*	* 605585	AR ?Pontocerebellar hypoplasia, type 15

Curiously, both patients with *NBAS* variants were heterozygous for the same variants despite them not being known to be related. The disease phenotype on both patients fitted the condition well. We were not able to confirm that they were compound heterozygous but there is a patient described in the literature who is reported to be compound heterozygous for the same variants [18].

Whole exome sequencing is not traditionally used to identify mitochondrial DNA variants despite mtDNA reads being generated. This is because standard pipelines do not report mtDNA variants. However, a number of studies have shown WES to be a valid tool for mtDNA analysis when the appropriate pipeline is used [19, 20]. The RD-Connect GPAP pipeline does allows, with some limitations, for the calling of mtDNA variants [10, 21]. As such, we were able to identify a variant in the mt-tRNA^Asn^ (*MT-TN*). This was classified by us as a variant of uncertain significance but was previously reported to be disease causing, with a phenotype similar to that of our patient, and so it remains a possible diagnosis.

A limitation of our study is that, because of the historic nature of the cohort, we were not able to confirm the variants using alternative methods, and we were not able to do segregation studies and confirm phase. Due to constraints of the GPAP and of WES we were not able to systematically look for structural variants or copy number variants.

## DISCUSSION

Reanalysis of exomes from 14 individuals with a suspected mitochondrial disorder, based on clinical, as well as histochemical and/or biochemical phenotype, detected a possible or likely diagnosis in 8 (57%). This included variants in mitochondrial DNA (*n* = 1, 12.5%), variants in nuclear encoded mitochondrial genes (*n* = 2, 25%) as well as variants in genes that cause other inborn errors of metabolism (*n* = 5, 62.5%). This highlights that systematic reanalysis of existing WES data is an important route for increasing the diagnostic yield in cohorts of patients with suspected mitochondrial disorders. WES reanalysis has been a valid method to increase diagnostic yield in a number of studies [10,22], and although there are challenges associated with data reanalysis (including time, manpower and cost), it should be considered before generating further genomic and other ‘omics’ data [10].

Additionally, taking an agnostic approach, rather than a gene panel approach, to the WES (and WGS) data analysis increases diagnostic yield in mitochondrial disorders [4, 8]. This is partly because of the heterogenicity of mitochondrial disorders themselves and the increasing role of ‘phenocopies’ in the diagnosis of patients with suspected mitochondrial disorders. Defects in mitochondrial respiratory chain complexes are typically an attribute of mitochondrial diseases and combined respiratory chain defects are common finding in disorders of mitochondrial energy metabolism [23]. None the less, in line with other published literature [24], 5 of our patients with combined respiratory chain defects had likely disease-causing variants in nonmitochondrial genes. Similarly, in a cohort study of patients with suspected mitochondrial disorders who underwent WGS, 63% of newly diagnosed patients had a nonmitochondrial diagnosis [8]. In line with this finding, in our cohort only 3 (38%) of the patients with a likely diagnosis found had a mitochondrial disease and in 5 cases (63%) we identified a nonmitochondrial diagnosis (*PNPLA2, CDC40, NBAS* and *SLC7A7* genes). Pathogenic variants in these genes all cause metabolic disorders with some clinical features that overlap with mitochondrial disease, and it is likely that biochemical evidence of respiratory chain deficiency is a secondary cellular process in other disorders [24]. Our study further highlights the advantages of ‘untargeted’ analysis.

Reanalysis of our cohort has lifted the diagnostic yield of the original cohort by 15%. Of note this cohort was extremely well characterized from a biochemical, histological, and clinical point of view and therefore the diagnostic yield is high both in the original paper (60%) [4] and our reanalysis (15%). In a more heterogeneous cohort of patients with suspected mitochondrial disorders the diagnostic yield following WGS was 31% [8]. Additionally, all individuals in our cohort (14/14) where children or young adults at the time of recruitment and or disease onset and it was previously noted that the diagnostic yield of children with suspected mitochondrial disorder by WGS was higher than that of adults [8].

Other important factors that are likely to have contributed to the relatively high diagnostic yield are: i) improved WES diagnostic pipeline provided by the RD-Connect GPAP [11]; ii) a number of variant interpretation resources have become available since the original cohort was published in 2014 including the ACMG guidelines for variant interpretation [15] as well as a number of additional tools for variant classification such as improved computational algorithms for predicting pathogenicity; iii) novel genes, with variants in two of the genes likely to be the disease causing in our cohort (*NBAS* and *CDC40*) being established as disease causing after the original cohort was published; iv) agnostic approach with manual curation of candidate variants using disease databases such as OMIM, and literature search.

The variants in *MT-TN*, *PDHA1* and *SLC7A7* in our cohort, as well as variants in *SUCLA2* and *PNPLA2* different to the ones identified in our cohort, were reported in the literature prior to the original Taylor *et al* cohort was published [4] but these were not identified in the paper as possible disease-causing variants. The *MT-TN* m.5698G > A variant is classified by us as a variant of uncertain significance and therefore, even if the variant was identified, the team may have wished to seek an alternate diagnosis by WES. With the regards to the *PDHA1*, *SLC7A7, SUCLA2* and *PNPLA2* variants, although we cannot be sure as we have no access to the original Taylor *et al*. pipeline and the raw data, it is possible that the variants were not called by the original pipeline and therefore not reported. In our cohort we did not identify any novel genes.

Despite careful WES data reanalysis and detailed phenotyping, in 6 of the 14 patients a diagnosis or likely diagnosis was not established. This included a patient who subsequently had trio WGS with a diagnosis or possible diagnosis still not identified. WES has limitations in the identification of structural variants, copy number variants, rare intronic variants and mitochondrial variants and it may also be that the causative genes have not yet been ‘discovered’.

Establishing a diagnosis is important for patients and their families and can potentially change clinical management [2]. Reanalysis of WES data has consistently shown to increase diagnostic yield in cohorts of undiagnosed patients with rare disease and periodic reanalysis of genomic data is important particularly when enough time has lapsed when technology is likely to have evolved and new genes possibly discovered [25–27]. With novel mitochondrial genes being discovered at a rate of about 20 per year since the advent of NGS [36], we suggest reanalysis every 2 years as suggested by the ACMG [25] could be an appropriate timeline. In our cohort all but one patient could have had a diagnosis after 2 years, if the GPAP pipeline was used. To overcome some of the issues associated with reanalysis, including cost, expertise and time required, some automation and machine learning algorithms may have an important role [10, 28]. Additional to WES and WGS, which further increases the diagnostic yield in patients with suspected mitochondrial disorders [8], new omic technologies such as long read sequencing, transcriptomics, proteomics and metabolomics are likely to be needed to improve diagnostic yield in similar cohorts. Deep phenotyping will continue to be key in all the additional techniques and for automation of analysis.

## Data Availability

Genetic and phenotypic data of the study participants has been uploaded to RD-Connect GPAP. The data supporting the findings of this study are available within the article and/or its supplementary material.
